# A novel kartogenin-platelet-rich plasma gel enhances chondrogenesis of bone marrow mesenchymal stem cells in vitro and promotes wounded meniscus healing in vivo

**DOI:** 10.1186/s13287-019-1314-x

**Published:** 2019-07-08

**Authors:** Feng Liu, Hongyao Xu, He Huang

**Affiliations:** 1 Department of Orthopaedics, The First Affiliated Hospital with Nanjing Medical University, 300 Guangzhou Road, Nanjing, 210029 Jiangsu China; 2 Department of Sports Medicine and Joint Surgery, Nanjing First Hospital, Nanjing Medical University, 68 Changle Road, Nanjing, 210006 Jiangsu China; 3 China Orthopaedic Regeneration Medicine Group, Zhejiang, 310000 Hangzhou China

**Keywords:** Kartogenin, PRP, BMSCs, Chondrogenesis, meniscus injury, Meniscus regeneration

## Abstract

**Background:**

The meniscus tear is one of the most common knee injuries particularly seen in athletes and aging populations. Subchondral bone sclerosis, irreparable joint damage, and the early onset of osteoarthritis make the injured meniscus heal difficultly.

**Methods:**

The study was performed by in vitro and in vivo experiments. The in vitro experiments were carried out using the bone marrow stem cells (BMSCs) isolated from the rabbits, and the stemness of the BMSCs was tested by immunostaining. The BMSCs positively expressed stem cell markers were cultured with various concentrations of kartogenin (KGN) for 2 weeks. The chondrogenesis of BMSCs induced by KGN was examined by histochemical staining and quantitative RT-PCR. The in vivo experiments were completed by a rabbit model. Three holes were created in each meniscus by a biopsy punch. The rabbits were treated with four different conditions in each group. Group 1 was treated with 20 μl of saline (saline); group 2 was treated with 5 μl of 100 μM KGN and 15 μl saline (KGN); group 3 was treated with 5 μl of 100 μM KGN, 5 μl of 10,000 U/ ml thrombin, and 10 μl of PRP (KGN+PRP); group 4 was treated with 10,000 BMSCs in 10 μl of PRP, 5 μl of saline solution, and 5 μl of 10,000 U/ml thrombin (PRP+BMSC); group 5 was treated with 10,000 BMSCs in 10 μl of PRP, 5 μl of 100 μM KGN, and 5 μl of 10,000 U/ml thrombin (KGN+PRP+BMSC). The menisci were collected at day 90 post-surgery for gross inspection and histochemical analysis.

**Results:**

The histochemical staining showed that KGN induced chondrogenesis of BMSCs in a concentration-dependent manner. The RT-PCR results indicated that chondrocyte-related genes were also increased in the BMSCs cultured with KGN in a dose-dependent manner. The in vivo results showed that large unhealed wound areas were still found in the wounds treated with saline and KGN groups. The wounds treated with BMSCs-containing PRP gel healed much faster than the wounds treated without BMSCs. Furthermore, the wounds treated with BMSCs-containing KGN-PRP gel have healed completely and formed more cartilage-like tissues than the wounds treated with BMSCs-containing PRP gel.

**Conclusions:**

BMSCs could be differentiated into chondrocytes when they were cultured with KGN-PRP gel in vitro and formed more cartilage-like tissues in the wounded rabbit meniscus when the wounds were treated with BMSCs-containing KGN-PRP gel. The results indicated that the BMSCs-containing KGN-PRP gel is a good substitute for injured meniscus repair and regeneration.

## Background

Meniscus is a rubbery tissue located between the femoral condyle and tibial plateau of the knee and aids in the force transmission, shock absorption, joint stability, lubrication, and proprioception of the knee joint [1–3]. The meniscus was dissected into inner and outer zones. The structure and composition of the two zones are different. The inner zone of the meniscus is an avascular/aneural region (white-white zone) with an articular cartilage-like structure, and the cells produce predominantly type II collagen and proteoglycans. The outer zone of the meniscus is a vascular/neural region (red-red zone) with a fibrocartilage-like structure and composed of cells with a higher proportion of type I collagen. These two areas are separated by the red-white region, which presents attributes from both red-red and white-white regions. The anatomical difference in the vascular supply limits the ability of meniscus to heal. It has been reported that the healing capacity of each area is directly related to blood circulation, leaving the white region susceptible to permanent post-traumatic and degenerative lesions [4].

Meniscus tears are very common injuries which occurred in athletes and aging populations [1]. If the injury happens at white-white zone or red-white zone, the injured meniscus heals poorly due to the lack of blood flow [5]. Currently, the injured menisci are treated surgically by partial or total meniscectomy [4]. Both procedures have shown poor long-term clinical results as evidenced by articular cartilage degeneration, articular surface flattening, subchondral bone sclerosis, irreparable joint damage, and the early onset of osteoarthritis [3]. The studies have found that meniscectomy can cause osteoarthritis [6, 7]. Although new techniques such as meniscal rasping and implantation of the synovial flap for enhancing the injured meniscus healing have been developed, the absence of healing, suboptimal repair outcomes with lengthy repair times were found in the inner avascular zone [1, 8]. Meniscal allograft transplantation (MAT) is considered a potential solution to restore knee biomechanics, improve clinical outcomes, and, possibly, delay the onset of knee osteoarthritis (OA) and has been widely used in recent years [9, 10]. However, there are still several controversial issues related to MAT. The chondroprotective effect of MAT is still not completely proven [11]. Furthermore, the failure of transplant is always occurred by immunoreactions, and the risk of disease transmission also limits allograft transplantations [12].

Tissue engineering is a newly emerging biomedical technology and methodology based on a smart and unique combination of cells, growth factors, and scaffolds to assist and accelerate regenerating and repairing of defective and damaged tissues [13, 14]. The animal experimental results have demonstrated that mesenchymal stem cell (MSC)-based tissue engineering technique provides a promising alternative for repairing injured meniscus due to their multi-differentiation potential [15, 16]. Using a stem cell-based approach to repair the damaged cartilage has shown promising results [17].

Recently, a small heterocyclic compound named kartogenin (KGN) was demonstrated to promote robust chondrocyte differentiation of the MSCs of humans [17], rabbits [18], and rats [19]. We also found that the KGN-treated autologous tendon graft can be used as a meniscal implant for meniscus regeneration [20].

Platelet-rich plasma (PRP) is an autologous plasma fraction containing high concentrations of platelets enriched with various growth factors such as platelet-derived growth factor (PDGF), transforming growth factor (TGF), and vascular endothelial growth factor (VEGF) and known to accelerate healing of tissues [21]. Several clinical studies have demonstrated that PRP injections have improved function and decreased pain to various maladies, including the elbow, wrist shoulder, hip, knee, ankle, plantar fascia, and meniscus [22–26].

There are two key considerations for repairing a damaged meniscus. The first consideration is that the defective area must have enough stem cells, and the second consideration is that the stem cells at the defective area must differentiate into cartilage cells. In order to ensure enough stem cells in the wound area, we injected rabbit BMSCs-containing PRP gel into the defect area. In order to induce the chondrogenic differentiation of these stem cells, we added KGN into the BMSCs-PRP gel to treat the wounded meniscus. Based on the previous studies, we hypothesize that BMSCs can be differentiated into chondrocytes when they are cultured with KGN-containing medium, and enhance meniscal regeneration in vivo when the BMSCs are injected into wounded meniscus with KGN-containing PRP gel together. To test our hypothesis, we isolated BMSCs from rabbit femur bones and determined the effect of KGN-containing PRP gel on the chondrogenic differentiation potential of the BMSCs in vitro, which enhanced the wounded meniscus healing in vivo.

## Materials and methods

Fifteen New Zealand white rabbits (6 months old, female) were used in this study. The experiments were done at Nanjing Medical University (NMU) following the approved protocol by the Institutional Animal Care and Use Committee (IACUC) of NMU.

### Isolation of bone marrow-derived stem cells (BMSCs)

Three rabbits were sedated by intra-muscular injection of ketamine (10 mg/kg) and xylazine (3 mg/kg), and then sacrificed with pentobarbital (120 mg/kg). The BMSCs were isolated from femur bones according to the published protocols [27]. Briefly, a needle (18-gauge) fastened to a syringe containing 0.2 ml of heparin (1000 units/ml) to aspirate 2 ml of bone marrow followed by washing the aspirates twice with phosphate-buffered saline (PBS). The bone marrow-PBS solution was centrifuged at 1500*g* for 5 min. After discarding the supernatant, the cells were resuspended in growth medium (20% fetal bovine serum in DMEM with 1% of penicillin and streptomycin) and incubated at 37 °C with 5% of CO_2_ and 95% of air atmosphere.

### PRP preparation

PRP was prepared from autologous blood of the same rabbit based on the previously published protocols [28]. The concentration of the platelet in the PRP was four times higher than that in the whole blood. The blood was collected atraumatically to avoid premature platelet activation by exposure to excessive shear forces.

**Fig. 1:**
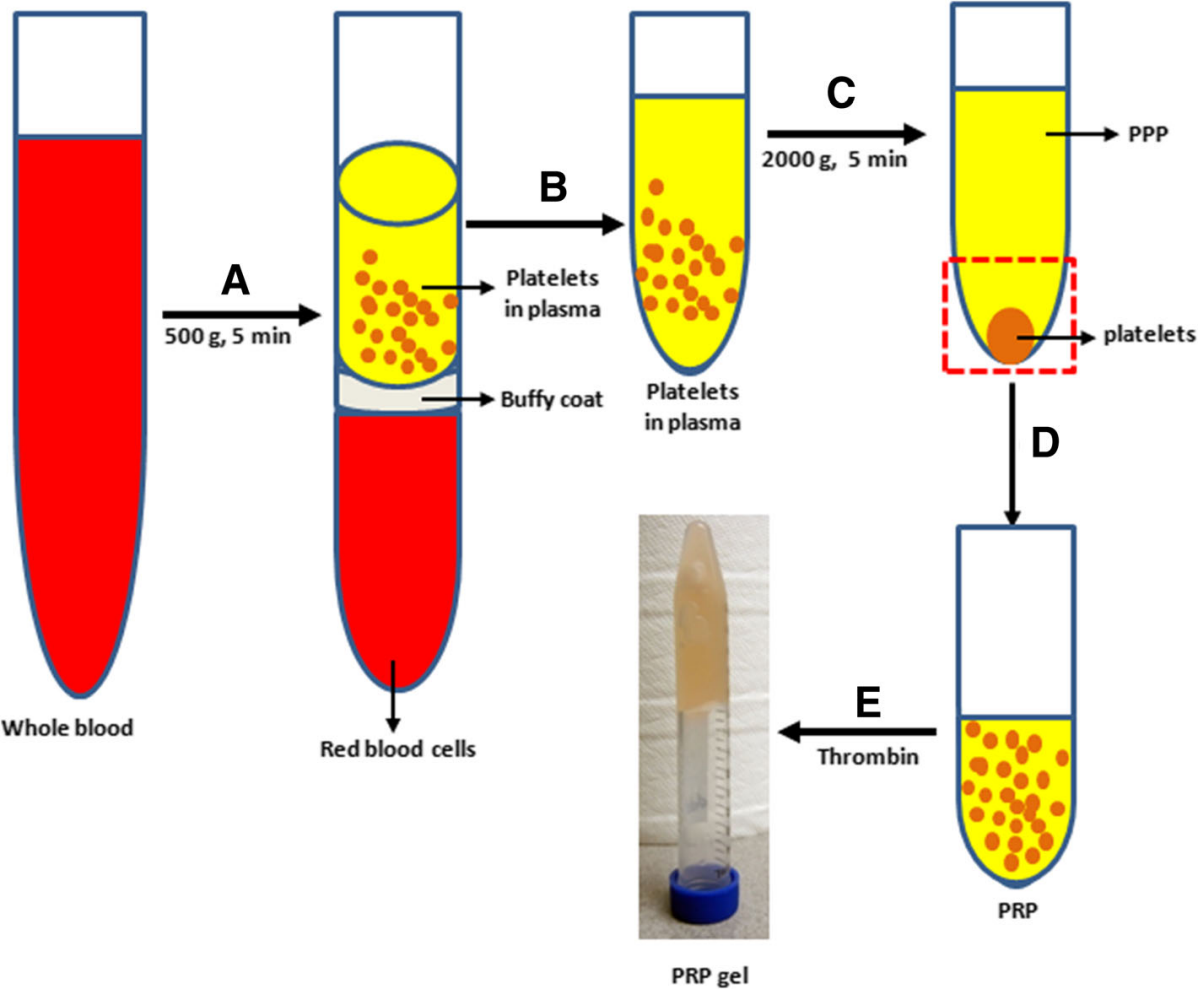
Preparation of platelet-rich plasma (PRP) from the whole blood by five steps. (**a**) Whole blood was separated into three layers by a centrifuge at 500*g* for 5 min. (**b**) The platelet-containing plasma (top layer) was transferred into a new centrifuge tube. (**c**) The platelet pallet was obtained by a centrifuge at 2000*g* for 5 min. (**d**) The PRP was prepared by suspending platelets with PPP. (**e**) The PRP gel was obtained by adding thrombin into PRP solution

Briefly, 9 parts of the whole rabbit blood were mixed with 1 part of 3.8% of sodium citrate (SC); the blood-SC mixture was separated into three layers by a centrifuge at 500*g* for 5 min (Fig. 1). The top layer was transferred into a new sterile tube and centrifuged at 2000*g* for another 5 min. The supernatant was collected and named as platelet-poor plasma (PPP). The platelet-containing pellet was suspended with appropriate volumes of PPP. The concentration of the platelet in the PRP was measured by automatic hematology analyzer (CELL-DYN Emerald, Abbott Laboratories, Chicago, IL, USA) and stored at 4 °C until further use.

### Characterization of rabbit BMSCs

The stemness of the BMSCs was examined by colony formation, multi-differentiation potential, and stem cell marker expression. The colony formation was examined by a microscope during the primary culture, and the formed colony was stained with methyl violet. The multi-differentiation potential of rabbit BMSCs was tested with adipogenesis, osteogenesis, and chondrogenesis. Briefly, the BMSCs at passage 1 were seeded in a 24-well plate at a density of 6 × 10^4^/well in basic medium (10% heated inactivated FBS, 100 U/ml of penicillin and 100 μg/ml of streptomycin in DMEM-low glucose) for 2 days. From the third day, some cells were still cultured with basic medium (control) and some cells were cultured either with adipogenic induction medium (Millipore, Cat. #SCR026, Billerica, MA, USA) for adipogenesis, or with osteogenic differentiation induction medium (Millipore, Cat. #SCR028, Billerica, CA, USA) for osteogenesis, or with chondrogenic induction medium (ThermoFisher, Cat. A1007101, Waltham, MA, USA) for chondrogenesis. After 3 weeks of culture, the adipogenesis was tested by oil red O staining, the osteogenesis was determined by alizarin red S staining, and chondrogenesis was tested by safranin O, alcian blue, and toluidine blue staining.

The stemness of the BMSCs was further tested by immunostaining on four stem cell markers: nucleostemin, stro-1, CD44, and CD90. The BMSCs at passage 1 were seeded in 12-well plates at the density of 3 × 10^4^/well and cultured with growth medium for 3 days. The cells were washed once with PBS fixed with 4% paraformaldehyde in PBS for 30 min at room temperature. For nucleostemin testing, the fixed cells were further treated with 0.1% Triton X-100 for 30 min and washed three times with PBS. The treated cells were incubated with goat anti-nucleostemin antibody (1:400, Neuromics, Cat. #GT15050, Edina, MN) at room temperature for 2 h. The cells were washed with PBS for three times and reacted with Cy-3-conjugated donkey anti-goat IgG antibody (1:500, Millipore, Cat. #AP180C, Billerica, MA) at room temperature for 1 h for nucleostemin testing.

For stro-1, CD44, and CD90 staining, the fixed cells were incubated either with mouse anti-stro-1 antibody (1:400, Invitrogen, Cat. #398401, Frederick, MD), or with rat anti-CD44 antibody (1:500, LifeSpan BioScience, Cat. LS-C13434-100, Seattle, WA, USA), or with mouse anti-CD90 antibody (1:400, antibodies online.com, Cat. #ABIN2472805) at room temperature for 2 h, and washed three times with PBS. The washed cells were reacted either with Cy3-conjugated goat anti-mouse IgG antibody (1:500, Millipore, Cat. #AP124C, Billerica MA) at room temperature for 1 h to test stro-1 and CD90 expression or with cy-3 conjugated goat anti-rat IgG antibody at room temperature for 1 h for CD44 testing.

The positively stained cells were examined under an inverted fluorescent microscope (Nikon Eclipse, TE2000-U) and analyzed by semi-quantification using SPOT™ imaging software (Diagnostic Instruments Inc., Sterling Heights, MI). Five views were taken from each well, and a total of 15 views from three wells were selected for each stem cell marker testing. The percentage of each stem cell marker expression was obtained by dividing the number of positively stained cells by the total number of the cells stained by the nuclear staining reagent Hoechst fluorochrome 33342 (1 μg/ml; Sigma, St. Louis, MO).

### Effect of KGN on chondrogenic differentiation of BMSCs in vitro

The KGN effect on chondrogenic differentiation of BMSCs was tested in vitro according to the published protocol [18]. The rabbit BMSCs were seeded with 0.5 ml of growth medium in 24-well plates at a density of 6 × 10^5^/well and centrifuged at 1500*g* for 5 min, and the formed cell pellet in each well was incubated overnight at 37 °C with 5% CO_2_. In the next morning, the medium was carefully removed, and the cell pallets were cultured with various concentrations of KGN-containing media (0, 10, 100, 1000 nM) for 2 weeks. The medium was changed every 3 days. At day 14, the cells were fixed with 70% ethanol in an ice bath for 1 h and washed with distilled water three times. The cells were then stained either with safranin O, or alcian blue, or toluidine blue for 1 h at room temperature according to the standard protocols. The stained cells were observed under an inverted microscope (Nikon Eclipse, TE2000-U), and a charge-coupled device camera was used to capture images and analyzed by imaging software (Diagnostic Instruments Inc., Sterling Heights, MI).

### Quantitative real-time RT-PCR for genetic analysis of BMSCs

The effect of KGN on chondrogenic differentiation of rabbit BMSCs was further analyzed by quantitative real-time reverse transcriptase-polymerase chain reaction (qRT-PCR) according to the published protocol [18]. After 2 weeks of culture with varying concentrations of KGN, the RNA was extracted from the BMSCs by using RNeasy Mini Kit (Qiagen). The gene expression in BMSCs was tested by two steps. At the first step, a total of 1 μg of RNA was used for first-strand cDNA synthesis by reverse transcription kit (Invitrogen). The synthesis of cDNA was performed at 65 °C for 5 min with cooling for 1 min at 4 °C, then 42 °C for 50 min and 72 °C for 15 min. At the second step, two chondrocyte-related genes, collagen type II and Sox-9, were tested by qRT-PCR using Qiagen QuantiTect SYBR Green PCR Kit (Qiagen) in a real-time PCR system (Step One Plus; AB Applied Biosystems). Glyceraldehyde-3-phosphate dehydrogenase (GADPH) was used as an internal control and the sequence. Rabbit-specific primers were designed according to the published method [28], and the sequences were listed as follows: 5′-TGG GTG TTC TAT TTA TTT ATT GTC TTC CT-3′ was used for collagen II (forward), 5′-GCG TTG GAC TCA CAC CAG TTA GT-3′ was used for collagen II (reverse); 5′-AGT ACC CGC ACC TGC ACA AC-3′ was used for SOX-9 (forward), 5′-CGC TTC TCG CTC TCG TTC AG-3′ was used for SOX-9 (reverse); 5′-ACT TTG TGA AGC TCA TTT CCT GGT A-3′ was used for GAPDH (forward), and 5′-GTG GTT TGA GGG CTC TTA CTC CTT-3′ was used for GAPDH (reverse). The PCR was performed for 50 cycles after an initial denaturation at 95 °C for 2 min. Each cycle needed a denaturation for 50 s at 95 °C with subsequent annealing for 50 s at 57 °C and extension for 40 s at 72 °C, and PCR reaction was terminated at 70 °C after a 10-min extension. Three independent experiments were performed to obtain a relative level of expression of the genes.

### In vivo meniscus repair experiment

Fifteen New Zealand white rabbits (6 months old, female) were used for in vivo experiment. The rabbits were anesthetized, and three holes were created in each meniscus using a biopsy punch (1 mm of diameter/hole). The rabbits were divided into five groups with three rabbits/group: the wounds in the saline group were treated with 20 μl of saline (saline); the wounds in the KGN group were treated with 5 μl of 100 mM KGN and 15 μl saline (KGN); the wounds in the KGN+PRP group were treated with 5 μl of 100 mM KGN, 5 μl of 10,000 U/ ml thrombin, and 10 μl of PRP (KGN+PRP); the wounds in the BMSC+PRP group were treated with 10,000 BMSCs in 10 μl of PRP, 5 μl of saline, and 5 μl of 10,000 U/ml thrombin (PRP+BMSC); the wounds in the KGN+PRP+BMSC group were treated with 10,000 BMSCs in 10 μl of PRP, 5 μl of 100 mM KGN, and 5 μl of 10,000 U/ml thrombin (KGN+PRP+BMSC). The menisci were collected at day 90 post-surgery for gross inspection and histochemical analysis.

### Histological analysis

At day 90 after surgery, menisci were harvested and placed in a pre-labeled base mold filled with frozen section medium (Neg 50; Richard-Allan Scientific; Kalamazoo, MI). The base mold with tissue samples was quickly immersed in liquid nitrogen cold 2-methylbutane and allowed to solidify completely. The tissue blocks were then either placed on dry ice and subsequently stored in a deep freezer (− 80 °C) until used for histological analysis or cut into 10-μm-thick sections, which were fixed with 4% paraformaldehyde for 30 min. The fixed tissue sections were stained with either hematoxylin and eosin (H&E) or safranin O and fast green according to standard protocols.

### Statistical data analysis

One-way ANOVA was used, then followed by Fisher’s PLSD test for multiple comparisons. When *P* values are less than 0.05, the two groups compared are considered to be significantly different.

## Results

**Fig. 2:**
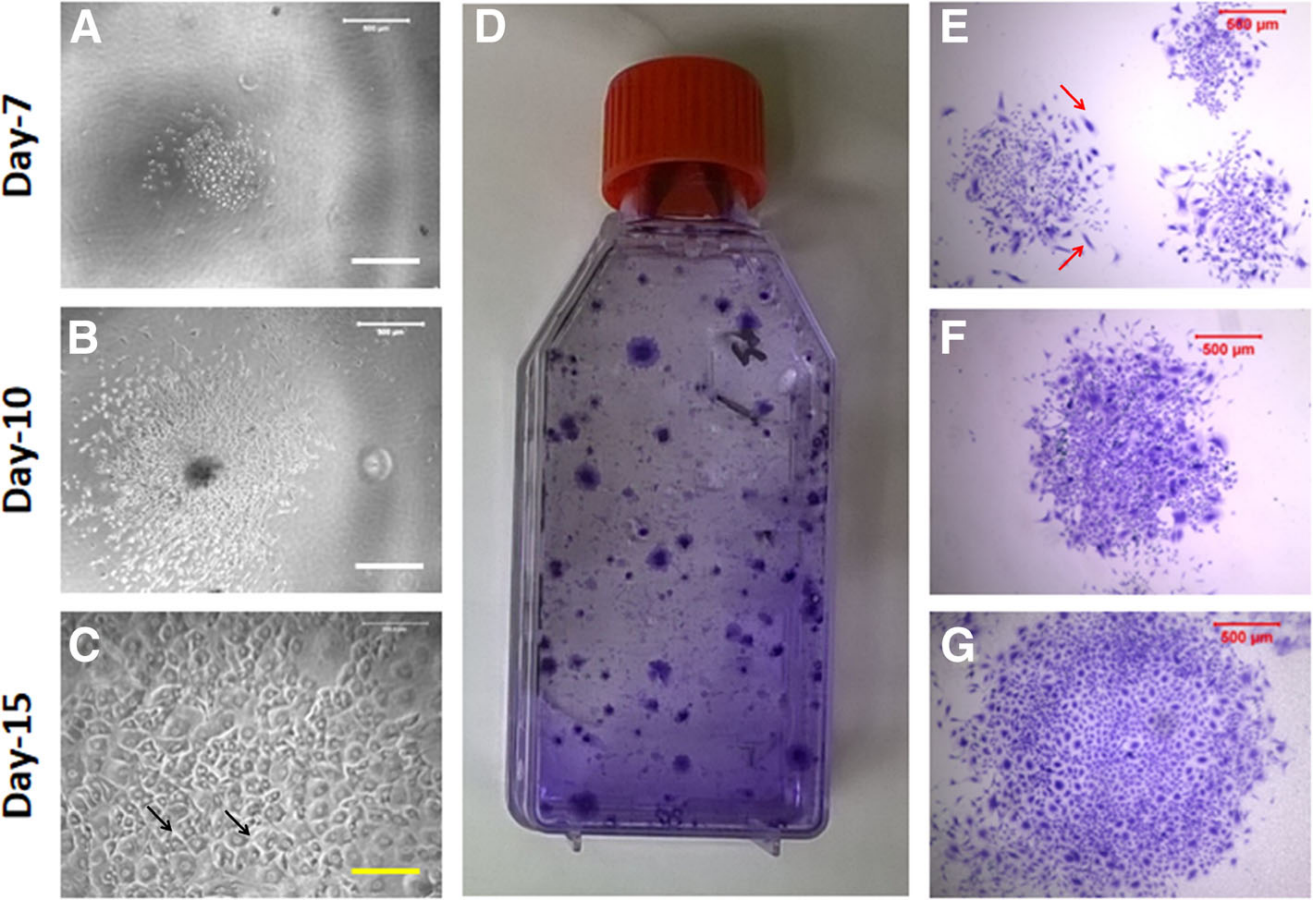
Colony formation of the BMSCs during primary culture. **a** A typical colony was formed at day 7. **b** A typical colony was formed at day 10. **c** A typical colony was formed at day 10. **d–g** The colonies were formed at day 15 and stained with methyl violet. The colonies formed by the BMSCs were heterogeneous as evidenced by cell shape and colony size (**d–g**). Some colonies were formed by cobblestone-like cells (black arrows in **c**), and some colonies were formed by spindle-like cells (red arrows in **e**)

Both in vitro and in vivo experiments first required the isolation of BMSCs from rabbit femur bones, and the stemness of these BMSCs was confirmed by colony formation, multi-differentiation potential, and stem cell marker expression. The results showed that the cells isolated from rabbit femur bone started the colony formation at day 3 of the primary culture (Fig. 2a) and the size of the colony increased during the culture (Fig. 2b, c). The methyl violet staining indicated that the colonies formed by rabbit BMSCs were heterogeneous (Fig. 2d–g). The most of BMSCs either were cobblestone shape (black arrows in Fig. 2c) or spindle shape (red arrows in Fig. 2e).

**Fig. 3:**
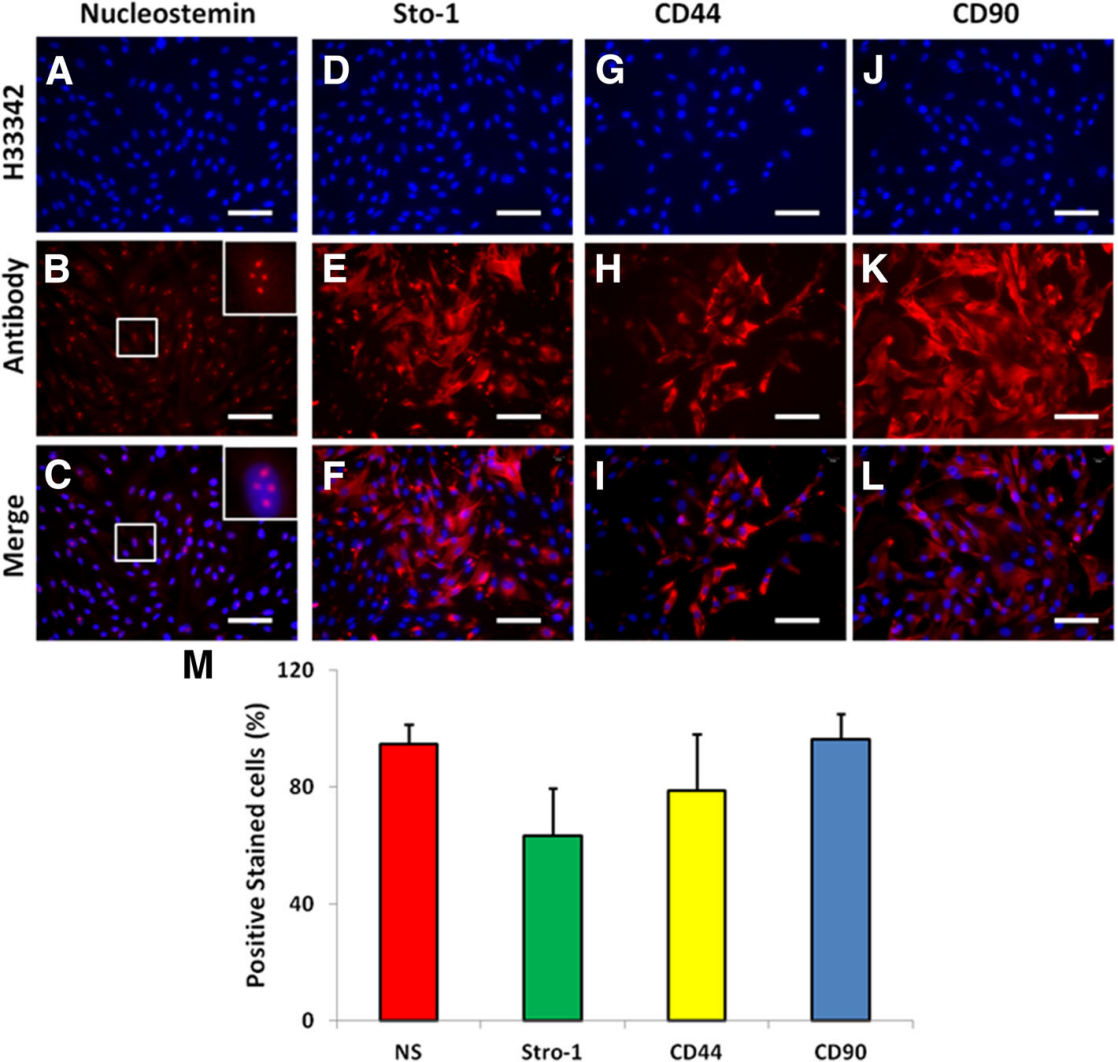
Stem cell marker expression in the BMSCs examined by immunostaining. **a**–**c** The cells were stained with nucleostemin. **d**–**f** The cells were stained with stro-1. **g**–**i** The cells were stained with CD44. **j**–**l** The cells were stained with CD90. **a**, **d**, **g**, **j** The cells were stained with H33342. **b**, **e**, **h**, **k** The cells were stained with the antibodies. **c**, **f**, **i**, **l** The images were merged images by **a**, **d**, **g**, **j**, and **b**, **e**, **h**, **k**. **m** The semi-quantification of positively stained cells by each stem cell marker. The insert images were enlarged box areas in the images of **b** and **c**. Bars 100 μm

Immunostaining indicated the cells isolated from the rabbit femur bones were stem cells as evidenced by more than 90% of BMSCs expressed nucleostemin (Fig. 3a–c, m), more than 63.43% of BMSCs expressed stro-1 (Fig. 3d–f, m), more than 78% of BMSCs were positively stained by CD44 (Fig. 3g–i, m), and more than 96% of BMSCs were positively stained with CD90 (Fig. 3j–l, m).

**Fig. 4:**
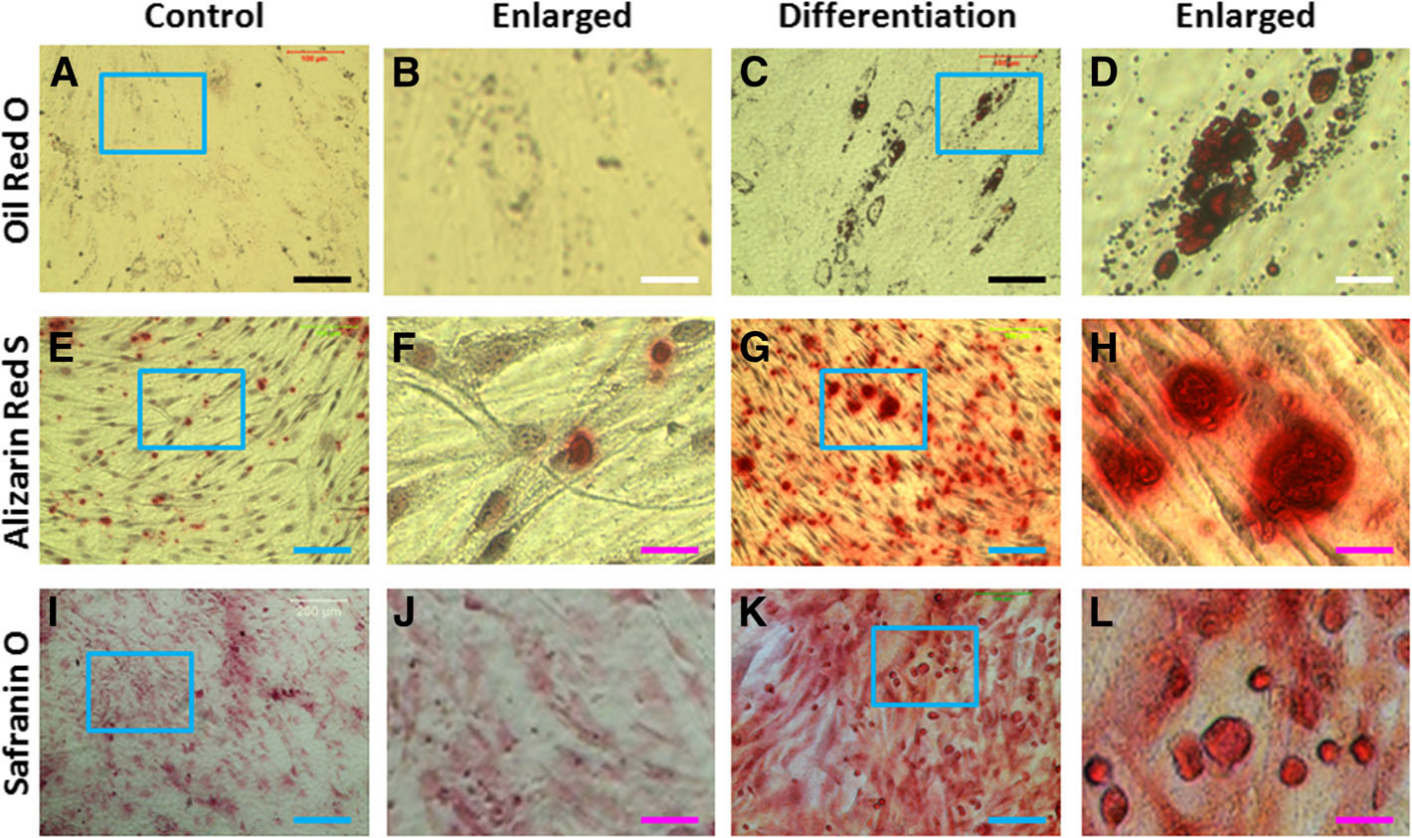
Multi-differentiation potential of the BMSCs cultured with various differentiation media for 3 weeks. **a**, **b** The cells were cultured with basic medium for 3 weeks and stained with oil red O. **c**, **d** The cells were cultured with adipogenic differentiation medium for 3 weeks and stained with oil red O. **e**, **f** The cells were cultured with basic medium for 3 weeks and stained with alizarin red S. **g**, **h** The cells were cultured with osteogenic differentiation medium for 3 weeks and stained with alizarin red S. **i**, **j** The cells were cultured with basic medium for 3 weeks and stained with Safranin O. **k**, **l** The cells were cultured with chondrogenic differentiation medium for 3 weeks and stained with Safranin O. Black bars 100 μm; white bars 20 μm; blue bars 200 μm; pink bars 40 μm

Further studies demonstrated that the cells isolated from rabbit bone marrow had multi-differentiation potential. After the BMSCs were cultured with various differentiation media for 3 weeks, they were positively stained either by oil red O (Fig. 4c, d), or by alizarin red S (Fig. 4g–h), or by safranin O (Fig. 4k–l). Lower percentages of the BMSCs grown in basic medium (control) were positively stained either with oil red O (Fig. 4a, b), or with alizarin red S (Fig. 4e, f), or with safranin O (Fig. 4i, j). These results indicated that the cells isolated from rabbit bone marrow were BMSCs and can be used for the following experiments.

**Fig. 5:**
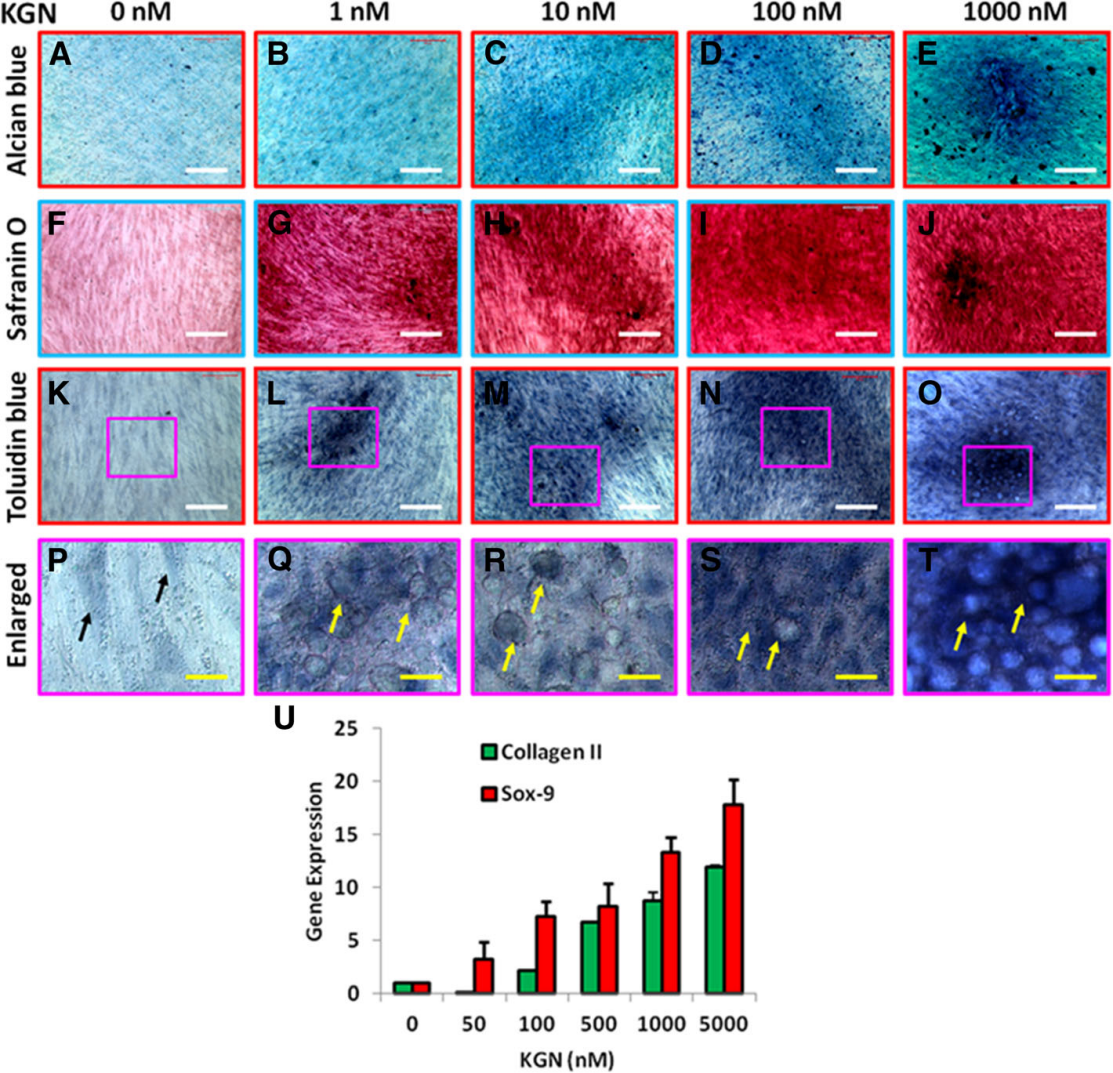
Chondrogenesis of the BMSCs induced by various concentrations of KGN for 2 weeks. **a**, **f**, **k**, **p** KGN concentration is 0 nM. **b**, **g**, **l**, **q** KGN concentration is 1 nM. **c**, **h**, **m**, **r** KGN concentration is 10 nM. **d**, **i**, **n**, **s** KGN concentration is 100 nM. **e**, **j**, **o**, **t** KGN concentration is 1000 nM. **a**–**e** The images were obtained by alcian blue staining. **f**–**j** The images were obtained by safranin O staining. **k**–**t** The images were obtained by toluidine blue staining. The images of **p**, **q**, **r**, **s**, and **t** were the enlarged box areas in the images of **k**, **l**, **m**, **n**, and **o**. All three stainings indicated that BMSCs were differentiated into chondrocytes induced by KGN in a concentration-dependent manner. KGN also induced morphology change of some BMSCs from spindle shape (black arrow in **p**) to round shape (yellow arrows in **q**–**t**). **u** The chondrocyte-related gene expression in the KGN-treated BMSCs examined by qRT-PCR. Both collagen II and Sox-9 gene levels were increased in the KGN-treated BMSCs in a concentration-dependent manner. White bars 100 μm; yellow bars 20 μm

More results showed that KGN induced the chondrogenetic differentiation of the BMSCs in a concentration-dependent manner (Fig. 5). The proteoglycan expression was increased in KGN-treated BMSCs as evidenced by three different chondrocyte staining reagents alcian blue (Fig. 5a–e), safranin O (Fig. 5f–j), and toluidine blue (Fig. 5k–t). The KGN-treated BMSCs also changed their morphology from spindle shape (black arrows in Fig. 5p) to round shape (yellow arrows, Fig. 5q–t) when they were cultured with KGN-containing medium. The qRT-PCR results indicated the upregulation of two chondrocyte-related genes, collagen II and SOX-9, found in the BMSCs cultured with KGN-containing medium in a concentration-dependent manner (Fig. 5u).

**Fig. 6:**
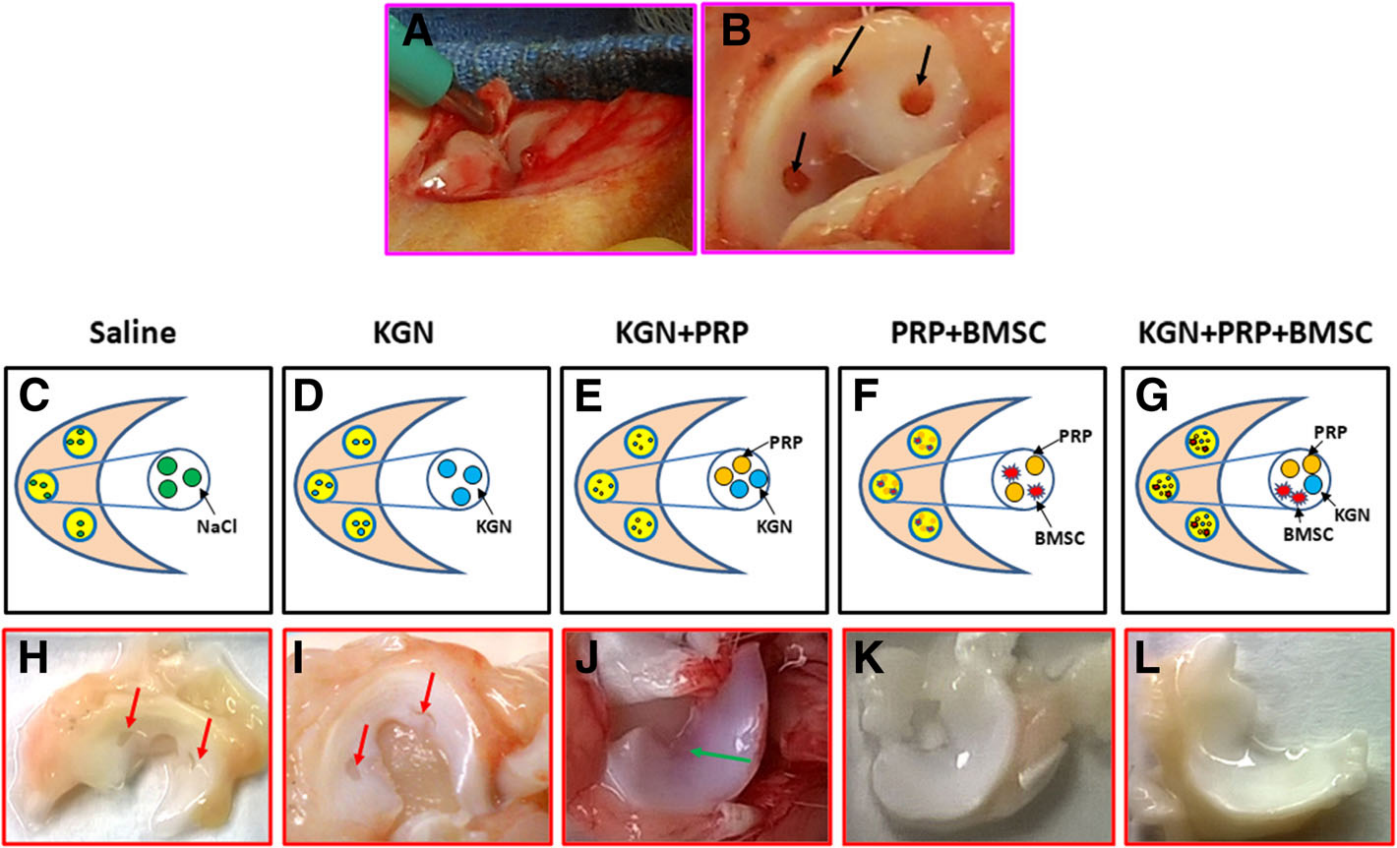
KGN effect on wounded meniscus healing by an in vivo rabbit model. **a** A biopsy punch with 1 mm diameter was used for creating a wound on rabbit meniscus. **b** Three holes (1 mm diameter/hole, black arrows in **b**) were created in each meniscus of 12 rabbits by a biopsy punch. **c** Each wound in three rabbits was treated with 20 μl of saline (saline). **d** Each wound in 3 rabbits was treated with 5 μl of 100 μMKGN and 15 μl of saline (KGN). **e** Each wound in 3 rabbits was treated with 5 μl of 100 μMKGN and 5 μl of 10,000 U/ml thrombin and 10 μl of PRP (KGN+PRP gel). **f** Each wound in 3 rabbits was treated with 5 μl of saline, 5 μl of 10,000 U/ml thrombin, 10 μl of PRP, and 10,000 BMSCs (PRP+BMSC). **g** Each wound in 3 rabbits was treated with 5 μl of 100 μMKGN, 5 μl of 10,000 U/ml thrombin, 10 μl of PRP, and 10,000 BMSCs (KGN+PRP+BMSC). **h** Gross view of the wounded rabbit meniscus at day 90 post-surgery with saline treatments. **i** Gross view of the wounded rabbit meniscus at day 90 post-surgery with KGN treatment. **j** Gross view of the wounded rabbit meniscus at day 90 post-surgery with PRP-KGN gel treatment. **k** Gross view of the wounded rabbit meniscus at day 90 post-surgery with BMSCs-containing PRP gel treatment. **l** Gross view of the wounded rabbit meniscus at day 90 post-surgery with BMSCs-containing KGN-PRP gel treatment. Large unhealed wound areas were still found in the saline-treated meniscus (red arrows in **h**) and KGN-treated meniscus (red arrows in **i**). Although the wounds were healed with KGN-PRP gels, the new formed meniscus tissue was thinner (green arrow in **j**) than BMSCs-containing PRP-treated wound (**k**) and BMSCs-containing KGN-PRP gel-treated wound (**l**). There was no unhealed wound area found in the meniscus treated with BMSCs-containing PRP gel and BMSCs-containing KGN-PRP gel and the new formed meniscus in both groups was thick and healthy (**k**, **l**)

Based on the above experimental results, we investigated the combination effect of BMSC-containing KGN-PRP gel on wounded meniscus healing using an in vivo rabbit model. Segregation of rabbits into 5 groups was done, and three holes were created in each rabbit meniscus by a biopsy punch (Fig. 6a, b). These wounded menisci were given different treatments namely saline (Fig. 6c), KGN (Fig. 6d), KGN+PRP (Fig. 6e), PRP+BMSC (Fig. 6f), and BMSC+KGN+PRP (Fig. 6g). Gross view of the meniscus at day 90 post treatments showed large unhealed wounded areas in the meniscus treated with saline (red arrows in Fig. 6h) and KGN (red arrow in Fig. 6i). The wounds healed to a good extent in the third group treated with KGN+PRP, but the meniscus tissue formed was thinner (green arrow in Fig. 6j) in comparison with the fourth group (Fig. 6k) and the fifth group (Fig. 6l). No unhealed wound areas were found in the meniscus treated either with BMSC+PRP (Fig. 6k) or with BMSC+KGN+PRP (Fig. 6l), and the new formed tissue was in normal thickness and healthy (Fig. 6k, l).

**Fig. 7:**
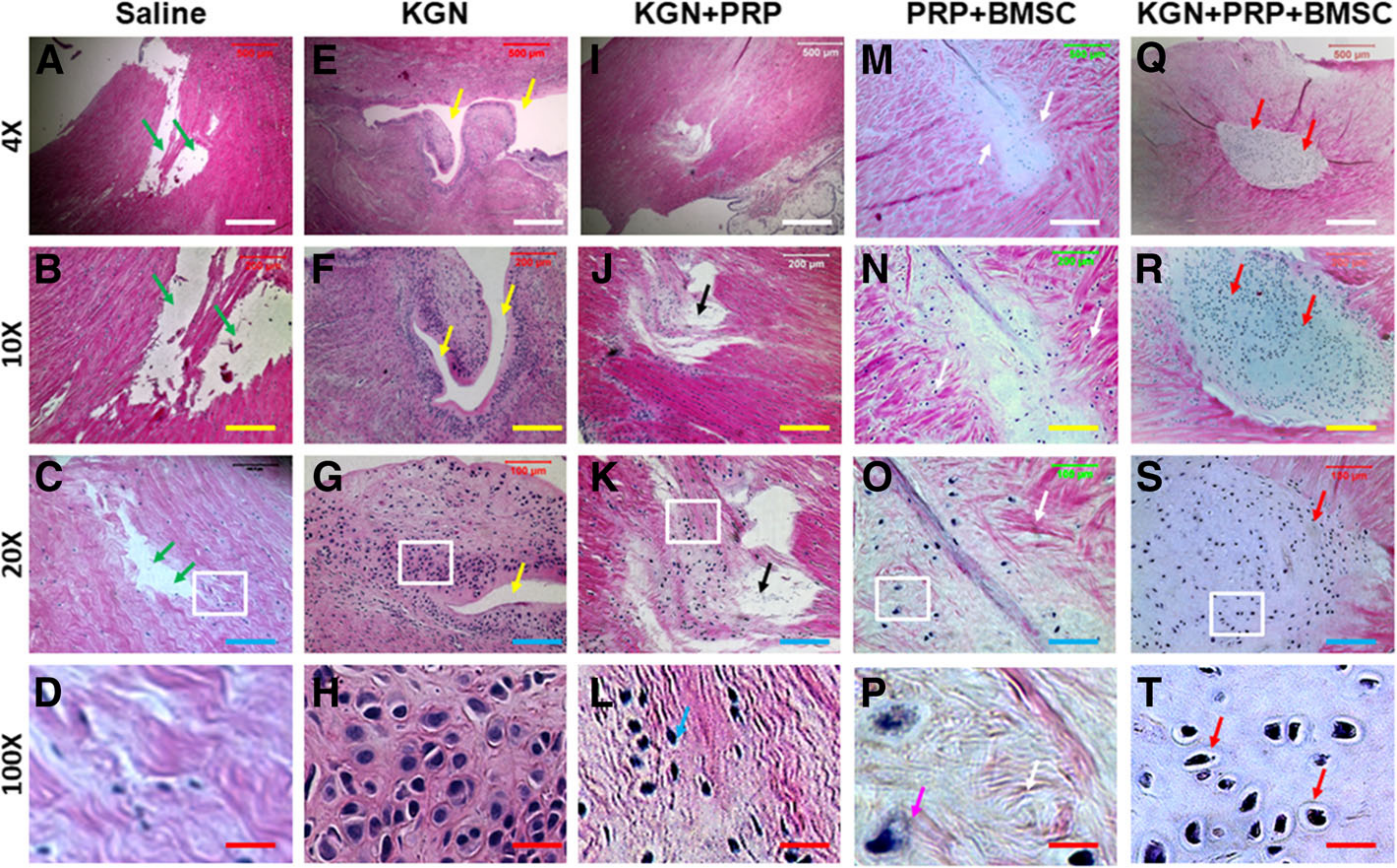
H&E staining of rabbit menisci after five different treatments for 3 months. **a**–**d** The wounded meniscus treated with saline at day 90 post-surgery. **e**–**h** The wounded meniscus treated with KGN at day 90 post-surgery. **i**–**l** The wounded meniscus treated with KGN-PRP gel at day 90 post-surgery. **m**–**p** The wounded meniscus treated with BMSCs-containing PRP gel at day 90 post-surgery. **q**–**t** The wounded meniscus treated with BMSCs-containing KGN-PRP gel at day 90 post-surgery. The images of **d**, **h**, **l**, **p**, and **t** were enlarged box areas in the images of **c**, **g**, **k**, **o**, and **s**, respectively. Large unhealed wound areas were still found in the saline-treated meniscus (green arrows in **a**, **b**, **c**) and KGN-treated meniscus (yellow arrows in **e**, **f**, **g**). Although the wounds were healed with KGN-PRP gels, new formed tissues were thinner than around tissues (black arrow in **i**, **j**, **k**) and less cells in the new formed tissues were fibrocartilage-like cells (blue arrow in **l**) than the wounds treated with BMSCs-containing PRP gel (**m**–**p**) and BMSCs-containing KGN-PRP gel (**q**–**t**). There was no unhealed wound area found in the meniscus treated with BMSCs-containing PRP gel (**m–p**) and BMSCs-containing KGN-PRP gel (**q–t**); however, the regenerated tissues were different in the wound areas treated with two different BMSCs-containing PRP gels. More collagen fiber-like tissues were found in the wound treated with BMSCs-containing PRP gel (white arrows in **m**–**p**), while more cartilage-like cells were found in the wound treated with BMSCs-containing KGN-PRP gel (red arrows in **q**–**t**). White bars 500 μm; yellow bars 200 μm; blue bars 100 μm; red bars 20 μm

**Fig. 8:**
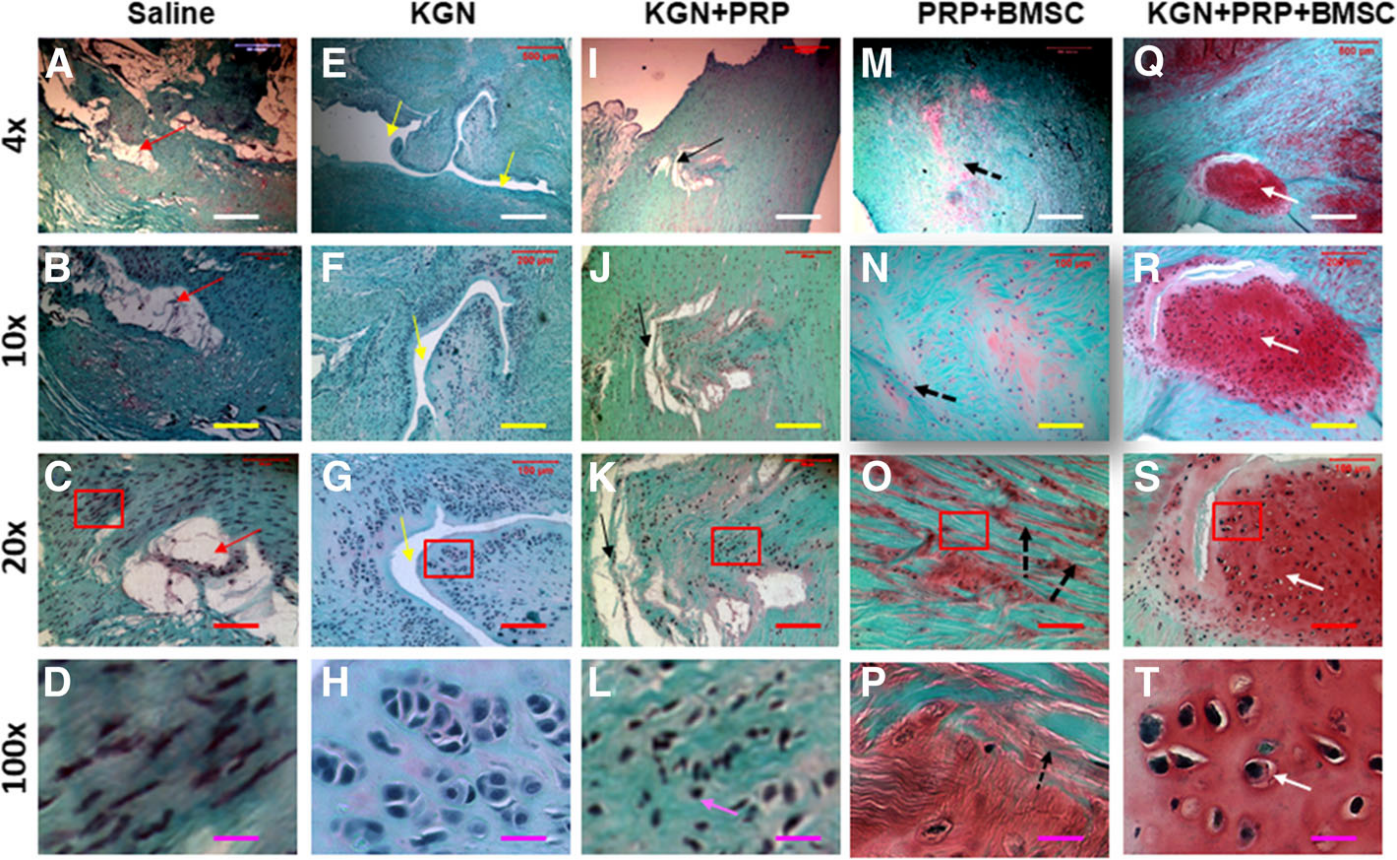
Safranin O and fast green staining of rabbit menisci after five different treatments for 3 months. **a**–**d** The wounded meniscus treated with saline at day 90 post-surgery. **e**–**h** The wounded meniscus treated with KGN at day 90 post-surgery. **i**–**l** The wounded meniscus treated with KGN-PRP gel at day 90 post-surgery. **m**–**p** The wounded meniscus treated with BMSCs-containing PRP gel at day 90 post-surgery. **q**–**t** The wounded meniscus treated with BMSC-containing KGN-PRP gel at day 90 post-surgery. The images of **d**, **h**, **l**, **p**, and **t** were enlarged box areas in the images of **c**, **g**, **k**, **o**, and **s**, respectively. Large unhealed wound areas were still found in the saline-treated meniscus (red arrows in **a**–**c**) and KGN-treated meniscus (yellow arrows in **e**–**g**). Although the wounds were healed with KGN-PRP gels, new formed tissues were thinner than around tissues (black arrows in **i**–**k**) and less cells in new formed tissues were fibrocartilage-like cells (pink arrow in **l**) than the wounds treated either with BMSCs-containing PRP gel (**m**–**p**) or with BMSCs-containing KGN-PRP gels (**q**–**t**). There was no unhealed wound area found in the meniscus treated either with BMSCs-containing PRP gel or with BMSCs-containing KGN-PRP gel, but the new formed tissues in these two groups were different, more collagen fiber-like tissues were found in PRP-BMSC-treated meniscus (black dash arrows in **m**–**p**), and more cartilage-like cells were found in the wound area treated with BMSCs-containing KGN-PRP gel (white arrows in **q**–**t**)

In addition, histochemical staining of the meniscus with H & E staining after BMSCs-containing KGN-PRP gel treatment (BMSCs+KGN+PRP) for 3 months showed the meniscus healed well and led to the formation of fibrocartilage-like tissue (Fig. 7q–t). Although the wounds treated with BMSCs-containing PRP gel healed completely, more collagen fiber-like tissues were found in the wound areas (Fig. 7m–p) than the wounds treated with BMSCs-containing KGN-PRP gel (Fig. 7q–t). There were large gaps found in the wound areas treated either with saline (Fig. 7a–d) or with KGN (Fig. 7e–h). Although the wounds were healed with KGN+PRP gel treatment (Fig. 7i–l), new formed tissue was thinner than the around tissues of the wound area (Fig. 7i–l). Furthermore, no cartilage-like tissue was found in the wound area treated with saline (Fig. 7a–d). These results were further demonstrated by safranin O and fast green staining (Fig. 8). The wound area treated with BMSCs+KGN+PRP was completely filled with fibrocartilage-like tissue as evidenced by positively stained cells with safranin O and fast green (red in Fig. 8q–t). Although the wounds treated with BMSCs-containing PRP gel (Fig. 8m–p) healed much better than the wounds treated either with saline (Fig. 8a–d) or with KGN+PRP gel (Fig. 8i–l), more collagen fiber-like tissues with less cartilage-like tissues were found in the wound areas treated with BMSCs-containing PRP gel (Fig. 8m–p) than the wounds treated with BMSCs-containing KGN-PRP gel (Fig. 8q–t). There were large unhealed wound areas that existed in the wounds treated either with saline (Fig. 8a–d) and KGN (Fig. 8e–h). The wounds treated with KGN+PRP showed some fibrocartilage-like tissues which were weakly stained with safranin O and fast green (Fig. 8i–l).

## Discussion

The aim of this study was to develop a novel bioactive scaffold for wounded meniscus healing. It is believed that such a scaffold should be structurally capable of supporting cell growth, promoting cell proliferation, enhancing vascularization, and inducing stem cell chondrogenic differentiation. To reach this aim, we developed this new bioactive scaffold using a rabbit BMSCs-containing KGN-PRP gel. Based on analysis and a thorough literature review on BMSCs and meniscal healing [17–20, 29, 30], we derived a hypothesis stating that BMSCs can form meniscus-like tissue when treated with KGN+PRP gel because (1) BMSCs when treated with KGN can proliferate and differentiate into chondrocytes in vitro and (2) treatment of BMSCs with KGN+PRP can enhance the healing of injured meniscus in vivo. To test our hypothesis, the experiments were performed using in vitro cell culture model and in vivo rabbit surgery model. The findings of this study established that KGN can induce differentiation of BMSCs into chondrocytes in vitro and form meniscus-like tissue in vivo. This was clearly evident from positive staining of BMSCs by safranin O, alcian blue, and toluidine blue when they were cultured with KGN-containing medium in vitro (Fig. 5), and histological staining of healed meniscus tissue sections by H&E and safranin O and fast green when the defective meniscus was treated with MSCs-containing KGN-PRP gel in vivo (Figs. 7 and 8).

It has been reported that the use of regenerative medicine in the treatment of wound healing can be applied through 1 of 3 main channels: injection of cells, scaffolds, and trophic factors [31]. Our results suggested that all three channels are important for wounded meniscus healing. The previous studies have shown that the BMSCs have a high potential of diverse chondrogenesis with the capacity to exhibit meniscus-like phenotype [32–36]. In the present study, we used rabbit BMSCs to promote defective meniscus regeneration. We found that stem cells play a critical role in wounded meniscus healing. Although a cell-free bioactive scaffold made by KGN and PRP could enhance the wounded meniscus healing, the healed meniscus was thinner than the host tissue (Fig. 6j) and the wounds treated with BMSCs-containing PRP gel (Fig. 6k) and BMSCs-containing KGN-PRP scaffold (Fig. 6l). Histological results showed that much less cartilage-like cells formed in the wound area treated with cell-free KGN-PRP scaffold than that formed in the wound areas treated either with BMSCs-containing PRP gel or with BMSCs-containing KGN-PRP scaffold (Figs. 7 and 8).

Our results have demonstrated that BMSCs play an important role in wounded meniscus healing. Our findings indicated that cartilage healing is mediated by bone marrow stem cells (BMSCs) and delivered by blood to the injured meniscus as occurs in other injured tissues [37]. The poor healing of injured meniscus is due to the poor blood supply to the “white-white” zone of the meniscus so the bone marrow-derived stem cells (BMSCs) fail to reach the injured meniscus resulting in its poor healing. To enhance the wounded meniscus healing, we delivered BMSCs within a PRP scaffold to ensure that the BMSCs could stay in the site of injury, and the fibrin of the PRP acts as a provisional matrix for cell proliferation and differentiation. It has been reported that the mesenchymal stem cells (MSCs) and bone marrow-mononuclear cells (BM-MNCs) are two important cell types used in cellular therapy. Since both have the same origin and pathway homing at the site of injury, the MSCs and BM-MNCs may belong to the same differentiation line, may have common cellular features and functions, and may have similar therapeutic efficacy. These cells have been shown to accelerate and promote the healing of various tissue injuries in animal and human studies [38]. Our results suggested that the treatment of BMSCs-containing KGN+PRP scaffold is a good approach for promoting the wounded meniscus healing.

Our results also indicated that in the healing process, some bioactive reagent, such as KGN is more important for cell proliferation and chondrogenic differentiation. Kartogenin is a small heterocyclic compound discovered recently, which is known to induce chondrogenic differentiation of BMSCs via core binding factor β (CBFβ)-runt-related transcription factor 1 (Runx1) pathway [17]. It has been reported that KGN was effectively used in repairing the damaged articular cartilage in mice with osteoarthritis [17]. Our results showed that large unhealed wound area was presented in the defective meniscus without KGN treatment (Figs. 6 7, and 8).

Moreover, our findings told that only KGN treatment without a carrier/scaffold was not good enough to enhance the wounded meniscus healing as evidenced by large unhealed wound areas found in KGN-treated wounded meniscus due to KGN flowing out (Figs. 6, 7, and 8). We found that the injection of KGN directly into the wound area could not keep enough concentration of KGN for wounded meniscus healing. In order to increase the efficacy of KGN treatment and avoid KGN flowing out, we used PRP gel as a KGN carrier. It is well known that PRP is a concentrated blood derivative that has been used to augment tissue healing in combination with tissue engineering modalities including BMSCs and tendon stem cells [39]. It stimulates chondrocytes to form cartilaginous tissue and enhance repair of meniscus defects [40]. In the present study, PRP gel not only carried the KGN in the wound area, but also enhanced the wounded meniscus healing by releasing some growth factors when the gel formation by adding bovine thrombin into the mixture of rabbit BMSCs, KGN, and PRP. Recent studies showed that PRP alone cannot effectively produce fibrocartilage zone [29, 30]. Our results also found that to achieve augmented results PRP should be combined with bio-compounds like KGN.

The first limitation of this study includes involvement of only 15 rabbits and we did not test BMSCs only group, PRP only group, and KGN+BMSCs group. In our future study, we will use more rabbits to complete these experiments including healing mechanism study and long-term benefits of KGN+PRP treatment. Secondly, the mechanical properties of the new formed meniscus were not tested; hence, future studies will focus on investigating the mechanical aspects of the regenerated meniscus. Thirdly, in the current study, we only used one ratio of KGN and PRP to treat the wounded meniscus, the regenerated tissue contained less collagen fibers than the normal fibrocartilage found in the rabbit meniscus. The previous study has shown that the concentration of PRP is important for tissue regeneration [41]. The future study will investigate the effect of different concentrations of KGN and PRP on fibrocartilage formation.

## Conclusion

Our findings indicate that KGN induces the differentiation of BMSCs into cartilage-like tissue in vitro and in vivo. Using PRP as a KGN and BMSC carrier promotes cartilage formation. Furthermore, the combination of BMSCs with KGN+PRP gel significantly enhanced cartilage-like tissue formation. Hence, we suggest that BMSCs-containing KGN+PRP gel may be used to accelerate and augment the repair of injured menisci in a clinical setting.

## Data Availability

For data requests, please contact the author.

## Abbreviations

BMSC: Bone marrow-derived stem cell
CBFβ: Core binding factor β
GADPH: Glyceraldehyde-3-phosphate dehydrogenase
H&E: Hematoxylin and eosin
KGN: Kartogenin
MSC: Mesenchymal stem cell
PBS: Phosphate-buffered saline
PDGF: Platelet-derived growth factor
PDT: Population doubling time
PPP: Platelet-poor plasma
PRP: Platelet-rich plasma
Runx1: Runt-related transcription factor 1
SC: Sodium citrate
SSEA-4: Stage-specific embryonic antigen 4
TGF: Transforming growth factor
VEGF: Vascular endothelial growth factor
